# Using value of information methods to determine the optimal sample size for effectiveness trials of alcohol interventions for HIV-infected patients in East Africa

**DOI:** 10.1186/s12913-018-3356-7

**Published:** 2018-07-31

**Authors:** Lingfeng Li, Jennifer Uyei, Kimberly A. Nucifora, Jason Kessler, Elizabeth R. Stevens, Kendall Bryant, R. Scott Braithwaite

**Affiliations:** 1 Department of Population Health, New York University School of Medicine, 227 East 30th Street, Floor 6, New York, NY 10016 USA; 2 National Institute on Alcohol Abuse and Alcoholism, National Institutes of Health, Bethesda, MD USA

**Keywords:** Value of information, Optimal sample size, Alcohol intervention, HIV, East Africa

## Abstract

**Background:**

Unhealthy alcohol consumption exacerbates the HIV epidemic in East Africa. Potential benefits of new trials that test the effectiveness of alcohol interventions could not be evaluated by traditional sampling methods. Given the competition for health care resources in East Africa, this study aims to determine the optimal sample size given the opportunity cost of potentially re-allocating trial funds towards cost-effective alcohol treatments.

**Methods:**

We used value of information methods to determine the optimal sample size by maximizing the expected net benefit of sampling for a hypothetical 2-arm intervention vs. control randomized trial, across ranges of policymaker’s willingness-to-pay for the health benefit of an intervention. Probability distributions describing the relative likelihood of alternative trial results were imputed based on prior studies. In the base case, policymaker’s willingness-to-pay was based on a simultaneously resource-constrained priority (routine HIV virological testing). Sensitivity analysis was performed for various willingness-to-pay thresholds and intervention durations.

**Results:**

A new effectiveness trial accounting for the benefit of more precise decision-making on alcohol intervention implementation would benefit East Africa $67,000 with the optimal sample size of 100 persons per arm under the base case willingness-to-pay threshold and intervention duration of 20 years. At both a conservative willingness-to-pay of 1 x GDP/capita and a high willingness-to-pay of 3 x GDP/capita for an additional health gain added by an alcohol intervention, a new trial was not recommended due to limited decision uncertainty. When intervention duration was 10 or 5 years, there was no return on investment across suggested willingness-to-pay thresholds.

**Conclusions:**

Value of information methods could be used as an alternative approach to assist the efficient design of alcohol trials. If reducing unhealthy alcohol use is a long-term goal for HIV programs in East Africa, additional new trials with optimal sample sizes ranging from 100 to 250 persons per arm could save the opportunity cost of implementing less cost-effective alcohol strategies in HIV prevention. Otherwise, conducting a new trial is not recommended.

## Background

Unhealthy alcohol consumption is common in East Africa [1] and multiple studies have shown that unhealthy alcohol consumption has exacerbated the HIV epidemic [2, 3]. For example, binge drinking and heavy alcohol consumption are associated with increased quantity of sexual partners, less likelihood of condom use, and increased commercial sex trade participation [4–8]. Furthermore, alcohol consumption also has a negative impact on adherence to antiretroviral therapies (ART) [7, 9–19]. Therefore, it is recommended that alcohol interventions that reduce harmful alcohol use among HIV infected patients in East Africa should be developed [3, 18], tested [19], and integrated as a part of HIV prevention and treatment programs [2, 6, 7, 20, 21], especially considering the fact that sub-Saharan African countries still account for almost 70% of new infections in the global HIV epidemic [22]. Even though a few alcohol interventions, such as brief interventions [23–27] and cognitive-behavioral therapy [28], have shown positive results in reducing alcohol consumption or increasing alcohol abstinence in prior studies, the prior evidence on the effectiveness of alcohol interventions may not be sufficient to eliminate decision uncertainty with regard to the implementation of such interventions among HIV-infected patients. Therefore, new trials to gain additional information/evidence on the effectiveness of alcohol interventions are likely necessary. Note that we define prior information or prior evidence as the effectiveness of an alcohol intervention studied in prior trials and define additional information or additional evidence as the information that will be concluded from new trials.

A new trial could benefit HIV prevention and treatment programs in East Africa by providing additional evidence on alcohol interventions and thereby reducing decision uncertainty regarding the implementation of alcohol interventions. However, traditional sampling approaches do not account for the potential benefit of such trials since they are primarily based on determining the minimum sample size required to detect the desired intervention efficacy for given Type I and Type II error probabilities [29, 30]. Value of information (VOI) methodology was introduced as an alternative to the traditional sampling methods based on the notion that new trial information is valuable because the benefit of generating additional evidence may exceed the opportunity cost of potentially re-allocating trial funds towards treatments with uncertain cost-effectiveness [31, 32], which is critical in resource-limited settings. Specifically, VOI estimates optimal sample size by maximizing the expected net benefit of sampling (ENBS), which is the difference between the expected value of sample information (EVSI) from a new trial and the cost of sampling. Applying the VOI method to identify optimal sample size is more favorable in literature when trial information could yield clinical actionable inferences, and this method should be considered in the early phase of trial design [29, 30, 33, 34]. For example, VOI calculations have been previously applied to determine the optimal sample size for future trials on catheter securement devices to inform the decision-making on the adoption of the devices [30]. Another advantage of VOI is that it could pre-determine the necessity of conducting a new trial before the sample size calculation for a new trial. VOI could provide an upper bound estimation on the potential benefit of a new trial by determining the expected value of perfect information (EVPI) [33, 35]. Therefore, conducting a new trial would be necessary only when EVPI is positive. For example, Micieli et al. used EVPI to quantify the uncertainty on the adoption of left atrial appendage occlusion devices over dabigatran or warfarin in atrial fibrillation and concluded that additional trials on the relative efficacy of stroke reduction between the two strategies would be necessary due to high EVPI [34].

Accordingly, the objective of this study was to use VOI methods to determine the necessity of a new clinical trial that aims to address the effectiveness of alcohol interventions for HIV-infected patients in East Africa and to identify the optimal sample size for such trials if the necessary condition is met.

## Methods

### Assumptions regarding hypothetical RCT

To be concordant with a prior high evidence level alcohol study among HIV-infected patients in East Africa [36] and four recent National Institute of Health(NIH) funded RCTs in East Africa [37–40], we studied a hypothetical randomized-controlled trial (RCT) that aimed to investigate the effect size of an alcohol intervention for HIV infected patients in East Africa with following assumptions: (1) Participants will be equally randomized to an intervention arm and a control arm; (2) Participant eligibility criteria are hazardous or binge drinkers (score ≥ 3 on the Alcohol Use Disorders Identification Test (AUDIT-C), a brief screening for heavy drinking and/or active alcohol abuse or dependence [36, 41–43]) and being ART-eligible or ART-initiated in the past 12 months; (3) The trial could be funded for 6–36 months; (4) Baseline trial costs, including program costs, marginal treatment costs, and reporting costs, were aggregated into a marginal cost per sample that was also based on the four RCTs (Table 1). Note that the primary interest of this work was to investigate evidence necessary to reduce uncertainty regarding the effect size of an alcohol intervention. The effect size was measured as the relative risk reduction of unhealthy alcohol consumption.

**Table 1:** Key model inputs

Variables	Baseline values	Sources
VOI variables		
Expected prior effect size^a^, $$ \overline{e} $$	45%	[28, 36, 45]
Sample size for the prior study, *n*_0_	75	[28, 36]
Marginal cost per sample^b^, $$ \overline{c} $$	$1140	[37–40]
HIV transmission variables		
Relative risk of unhealthy alcohol use on risky sex	1.29	[45]
Relative risk of unhealthy alcohol use on STIs	1.72	[45]
Relative risk of unhealthy alcohol use on ART non-adherence	2.33	[45]

*STIs* Sexually Transmitted Infections

^a^Effect size was measured as relative risk reduction due to the implementation of an alcohol intervention for unhealthy alcohol drinkers in population

^b^This summarizes program cost, treatment cost, and other costs

### Integrating prior information with new trial information: a Bayesian procedure

Prior information provided by the alcohol trials that test the effect size of an alcohol intervention is defined as the information already available before a new trial, which may not be sufficient to support optimal decision-making. We specified the certitude regarding an alcohol intervention’s effect size prior to additional information (e.g., “prior distribution”) based on results of Papas and colleagues who evaluated a cognitive behavioral therapy-based intervention for HIV-positive outpatients with unhealthy alcohol use in Kenya [28, 36]. The intervention was based on social cognitive theory [44], and was designed to increase alcohol abstinence by teaching skills to mitigate substance use circumstances caused by stress or other problems. The intervention had been proven effective during the treatment and follow-up phases in Papas’ study, and effect size information reported in the study was used to estimate the prior distribution below. Specifically, in prior distribution, *e*_0_ is the intervention effect size sampled from the prior beta distribution; $\overline{e}$is the expected prior effect size; and *n*_0_ is sample size of the prior study (Table 1).

Prior distribution:$$ {e}_0\sim Beta\left({n}_0\overline{e},{n}_0\left(1-\overline{e}\right)\right). $$

If a new trial with sample size *n* is initiated to gain additional information about the intervention effect size, in order to predict the sample statistics *α*_*D*_ for the new trial, we used a conjugate pair of the prior distribution to construct a predictive distribution. The predictive distribution is summarized below.

Predictive distribution: *α*_*D*_~*Binomial*(*e*_0_, *n*)

In order to utilize both of the prior and the new information on intervention effect sizes, an updated distribution was obtained by integrating the prior distribution and the predictive distribution through Bayesian Updating. *e*_1_ is updated effect size.

Updated distribution: $$ {e}_1\sim Beta\left({n}_0\overline{e}+{\alpha}_D,{n}_0+n-{n}_0\overline{e}-{\alpha}_D\right) $$

Given a specific simple size *n* for the new trial, we ran *I* iterations to generate statistics ${\alpha}_D^{(i)}\ \left(i=1,\dots, I\right)$. For each ${\alpha}_D^{(i)}$, we ran *J* iterations to generate ${e}_1^{(j)}\mid {\alpha}_D^{(i)}\ \left(j=1,\dots, J\right)$. We used a total number of one million (*I* × *J*) iterations to output a set of updated effect sizes to ensure the robustness of our VOI results. More details about the approach can be found elsewhere [31, 32].

### Incorporating the integrated information into a decision model of HIV treatment and prevention strategies

As previously discussed, alcohol interventions among HIV infected patients might reduce HIV transmission and improve health, and additional information on the effectiveness of such interventions comes with the opportunity cost of not spending those funds on the interventions themselves, which are potentially cost-effective. We used a previously published and validated HIV model to test the impact of the updated distribution of intervention effect size *e*_1_ on the decision of adopting (*s* = 1) versus not adopting (*s* = 0) the alcohol intervention [45, 46]. The model outcomes necessary for the subsequent cost-effectiveness evaluations and VOI calculations were quality-adjusted life years *QALY*(*s*, *e*_1_) and costs *cost*(*s*, *e*_1_) for the intervention scenario (*s* = 1) and the null scenario (*s* = 0) respectively. A detailed description of the HIV model is reported elsewhere [45, 46]. Briefly, the HIV model contains an HIV progression module and an HIV transmission module. The HIV progression module approximates the health outcomes by evaluating the change of CD4 cell counts and HIV-1 viral load for each HIV-infected individual. This module also simulates the effect of ART and takes the major causes of ART failure into account, such as ART non-adherence, non-adherence related genotypic resistance, and medication toxicity [46]. The individual-based HIV progression module interacts with a population-based compartmental HIV transmission module which simulates heterosexual transmission among the population in East Africa. The model compartments are differentiated based on health characteristics as well as behavioral risk characteristics. A hypothetical population switches compartments based on change to their health and behavior status. Probability of transmission is a function of multiple factors, including rate of acquiring new partners, duration of partnership, frequency of sexual contact within a partnership, and likelihood of condom use. The people who are in the compartments of unhealthy alcohol consumption were modeled as having these three major factors: increased risk of condom nonuse, increased risk of ART non-adherence, and increased STI prevalence (Table 1). The alcohol intervention reduces the HIV infection rate by transferring people from the compartments representing unhealthy alcohol consumption to those without unhealthy alcohol consumption.

### Assessing the value of information (VOI) added by a RCT

In value of information (VOI) methods, expected value of perfect information (EVPI) represents an upper bound estimate on the potential benefit of a new trial if we assume new trial information would be perfect to help policymakers eliminate all decision uncertainty. Therefore, EVPI is used in economic evacuation to determine the necessity of conducting a new trial. Mathematically, it is the difference between the value of the decision made based on perfect information (e.g., after an RCT without bias and with infinite sample size) and the value of the decision made based on prior information (e.g., before a RCT) [31, 32]:

$$ EVPI={E}_{e_1}{\mathit{\max}}_{\boldsymbol{r}} NB\left(s,{e}_1\right)-{\mathit{\max}}_r{E}_{e_1} NB\left(s,{e}_1\right) $$

A positive EVPI is a necessary condition for conducting a new RCT. Net benefit was calculated using the *QALYs* (*s*, *e*_1_) and *costs* (*s*, *e*_1_) generated by the HIV model using the following equation [31, 32]:

$$ NB\left(s,{e}_1\right)= QALY\left(s,{e}_1\right)\times WTP- cost\left(s,{e}_1\right) $$

WTP is the decision maker’s willingness-to-pay for incremental health benefit. The World Health Organization’s (WHO) Choosing Interventions that are Cost-Effective (CHOICE) program recommends benchmarking willingness-to-pay (WTP) based on gross domestic product (GDP) per capita [47], in particular between 1 and 3 times the annual GDP per capita. Alternatively and in greater accord with economic theory, WTP may be inferred from a desired program that is not fully implemented because of resource constraints (e.g., routine viral load testing).

While EVPI estimates an upper bound benefit when information is perfectly known after a new trial (i.e., sample size of the new trial approaches infinity), expected value of sample information (EVSI) estimates the potential benefit of a new trial with a finite sample size. We then calculated the expected value of sample information (EVSI) which compares the net monetary benefit of a decision made with the updated information (e.g. after the RCT) and the benefit of a decision made with the prior information (e.g., before the RCT):

$$ EVSI={E}_{\alpha_D}\mathit{\max}{E}_{e_1\mid {\alpha}_D} NB\left(s,{e}_1\right)-\mathit{\max}{E}_{e_1} NB\left(s,{e}_1\right) $$

${E}_{\alpha_D}\mathit{\max}{E}_{e_1\mid {\alpha}_D} NB\left(s,{e}_1\right)$ is the expected net benefit of the decision made based on the updated information (e.g., after the RCT), and $\mathit{\max}{E}_{e_1} NB\left(s,{e}_1\right)$ is the expected net benefit of the decision made with the prior information (e.g., before the RCT) [31, 32].

In additional to evaluating expected trial benefit, costs associated with conducting such a trial should be weighed against potential benefit in trial design. Expected net benefit of sampling (ENBS) values the expected net benefit of a new trial as the difference between the monetized expected value of sample information (EVSI) and the marginal investment of such a trial [31, 32]:

$$ ENBS= EVSI- CS= EVSI-\overline{c}\bullet n $$

In the ENBS equation, CS is cost of sampling; $\overline{c}$ is marginal cost per sample (Table 1); and *n* is the sample size of a trial. As sample size grows larger, the incremental certitude from information represented by EVSI grows smaller (i.e., diminishing returns) whereas the incremental cost of conducting the trial may not. As long as EVSI, or the value of conducting the new RCT, exceeds the corresponding study cost (ENBS > 0), the return on investment would be positive. An optimal sample size could be obtained at which the expected net benefit of sampling (ENBS) is maximized.

### Sensitivity analysis

Since ENBS is greatly influenced by policymakers’ willingness-to-pay (WTP), we performed a sensitivity analysis to evaluate how optimal results change over a series of WTP benchmarks, where WTP was measured using the standard metric of US$ per additional quality-adjusted life year (QALY), where QALY is a measure that aggregates additional quality and quantity of life. We also performed a sensitivity analysis across three intervention duration scenarios: long, medium, and short (20 years, 10 years, and 5 years respectively).

## Results

### Cost-effectiveness acceptability based on prior information

**Fig. 1:**
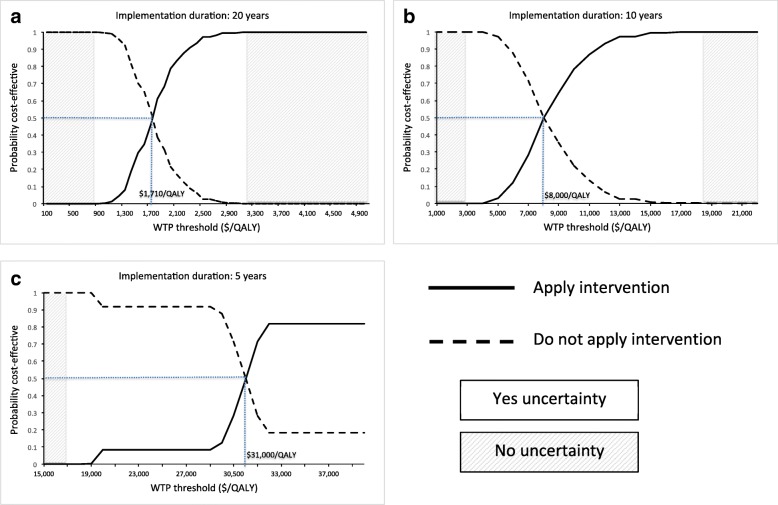
Cost-effectiveness acceptability curves when strategy implementation durations were 20 (**a**), 10 (**b**), and 5 years (**c**)

Given QALY and cost outcomes estimated by the HIV model, we applied cost-effectiveness acceptability curves [48] to identify where the decision uncertainty was greatest and how it varied by willingness-to-pay threshold. In Fig. 1a, when WTP threshold was greater than $3200/QALY or smaller than $900/QALY (shaded area), there would be no decision uncertainty with regard to choosing a more cost-effective strategy: prior evidence suggested that policymakers should adopt the intervention when WTP > $3200/QALY or should not adopt the intervention when WTP < $900/QALY. In Fig. 1a, decision uncertainty existed in the unshaded area, and the greatest decision uncertainty arose when WTP = $1710/QALY. In this case, additional evidence from a new trial might be necessary to increase the certitude. Similarly, if we implement a study for 10 or 5 years (Fig. 1b and c), the greatest uncertainty exists when WTP = $8000/QALY or $31,000/QALY respectively. Given the distributions of decision uncertainty in unshaded areas in Fig. 1, EVPI can be used to quantify the consequence of the decision uncertainty (or the health benefit of addressing the uncertainty) in a monetary format.

### Expected value of perfect information

**Fig. 2:**
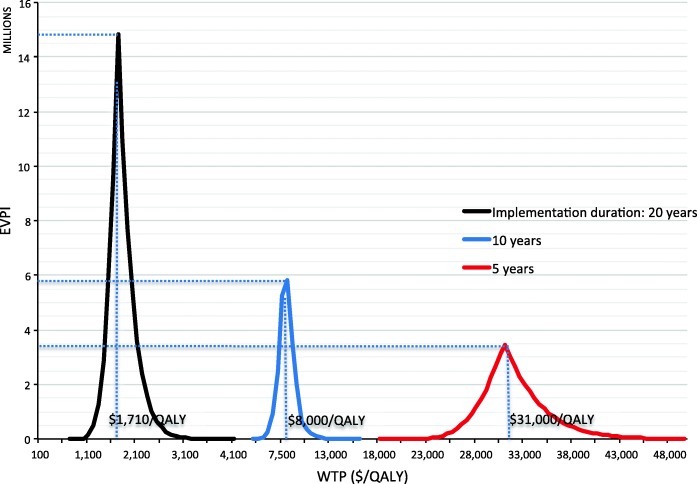
EVPI curves when the alcohol intervention durations were 20, 10, and 5 years

We used EVPI (Fig. 2) to quantify the uncertainties in Fig. 1, and the EVPI varied substantially over different WTPs and different durations of intervention implementation. When implementation duration was assumed to be 20 years, EVPI was maximized ($14.8 million) at a WTP of $1710/QALY, well within the suggested range of WTPs for East Africa ($1014/QALY to $3042/QALY), suggesting that a new RCT could yield valuable information. However, at shorter durations, the minimum WTP required to produce a positive EVSI fell outside the recommended range rendering the prospect of conducting a trial too expensive and not a good return on investment. For a duration of 10-years, EVPI was positive when WTP was greater than $4500/QALY and maximized ($5.8 million) at a WTP of $8000/QALY. For a duration of 5-years EVPI was positive when WTP was greater than $18,000/QALY and maximized ($3.4 million) at a WTP of $31,000/QALY. Since EVPI is the upper bound estimate on the potential benefit of conducing a new trial, our EVPI results in Fig. 2 indicated that it might be worthwhile to conduct a new trial when the intervention implementation duration is 20 years. However, when intervention duration was set to 10 or 5 years, EVPI was zero across the WHO recommended range of WTPs for East Africa ($1014/QALY to $3042/QALY) [47], suggesting that a RCT should not be conducted under these two scenarios.

### Optimal sample size estimation - base case

**Fig. 3:**
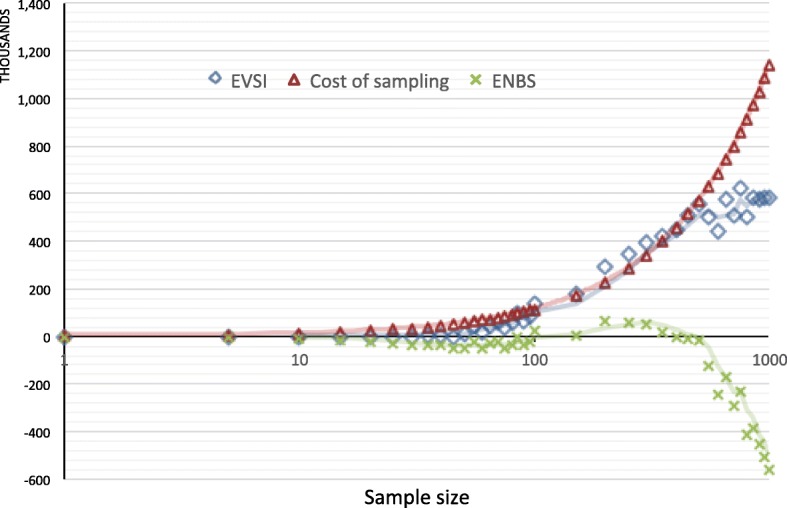
EVSI, ENBS, and cost of sampling curves for the base case

EVSI and ENBS were calculated to estimate an optimal sample size for a new study after the necessary condition was satisfied (EVPI > 0). At baseline intervention duration of 20 years and baseline WTP equal to $2473/QALY, the incremental cost per QALY of implementing routine viral load testing for HIV-infected patients in East Africa, incremental cost of sampling for the trial grew larger whereas EVSI produced by the trial was diminished as the sample size of the trial grew larger in Fig. 3. Thus, the optimal sample size was found at the place where ENBS was maximized in Fig. 3. Specifically, the optimal sample size for the new RCT was 200 (100 per arm) and the corresponding maximum ENBS was $67 thousand US dollars (Fig. 3) for the base case scenario.

### Optimal sample size estimation - sensitivity analysis

For a scenario of using a conservative WTP (1 X GDP/capita of East Africa, $1014/QALY) and assuming an implementation duration of 20 years (Table 2), conducting a new trial was not recommended since the return on investment was negative (ENBS < 0). Conducting a new trial was also not suggested even when policymakers were willing to pay more for an additional health gain and thereby using an upper bound WTP of 3 X GDP/capita ($3042/QALY) and also assuming an implementation duration of 20 years (Table 2). However, setting WTP equal to the incremental cost-effectiveness ratio (ICER) of the alcohol intervention ($1710/QALY, within the lower and upper WTP bounds but distinct from our base case assumption), ENBS rose to more than $11 million and corresponding optimal sample size reached 500 (250 per arm).

**Table 2:** Optimal sample sizes and maximum ENBS values for WTP benchmarks

Scenarios				Outcomes	
WTP value ($/QALY)	WTP benchmark	Source	Intervention duration (years)	Optimal sample size (each arm)	Maximum ENBS
$1014	1 x GDP/capita	[49]	20	NA^a^	< 0
$1124	HIV laboratory monitory strategy if two ART regimens are available	[46]	20	NA^a^	< 0
$1770	ICER of 20 year alcohol intervention	–	20	500	$11,309,000
$2473	HIV laboratory monitory strategy if three ART regimens are available (base case)	[46]	20	200	$ 67,400
$3042	3 x GDP/capita	[49]	20	NA^a^	< 0
$8000	ICER of 10 year alcohol intervention	–	10	500	$5,108,000
$31,000	ICER of 5 year alcohol intervention	–	5	240	$2,949,000

^a^Since return on investment for a new trial was negative (ENBS < 0), conducting a new trial was not recommended for this scenario

## Discussion

Our results suggest that new RCTs to test interventions aimed at increasing alcohol abstinence among HIV-infected patients in East Africa are worthwhile investments assuming that policymakers intend to implement the intervention for a longer duration. Under such circumstances and assuming a WTP of a simultaneously resource-constrained priority (routine virological testing for HIV-infected patients), a new RCT with the optimal sample size of 200 (100 per arm) would yield an expected net benefit of $67 thousand for East Africa.

When the alcohol intervention was assumed to be implemented for 10 or 5 years, an additional RCT would not yield information of favorable value across a plausible range of WTPs, and therefore our analyses suggest that it should not be conducted. In these scenarios, decision uncertainty was limited and standard care was always more cost-effective than the alcohol intervention given resource constraints in East Africa.

Notably, WTPs higher than the plausible range would result in RCTs having favorable value even with implementation durations of 10 years or 5 years. It is important to be mindful of this because even though the intervention duration is shorter, the opportunity cost of choosing a less cost-effective decision could still be high if decision makers are willing to pay a higher price for a QALY gain from the intervention.

The maximum ENBS for the intervention durations of 20 years, 10 years, and 5 years are $11 million, $5 million, and $3 million respectively (Table 2), illustrating that the longer implementation of the alcohol intervention could result in far greater health gains. This is because (1) The benefit of the alcohol intervention is unlikely to be fully captured at a population level if the intervention implementation duration is short; and (2) The selection of other HIV strategies, other than the alcohol intervention, could result in more health gains in an East African population given the same healthcare research budget.

This study has several limitations. First, even though our VOI analyses and HIV simulation model are robust, there is still a stochastic noise that cannot be eliminated due to the considerable computational complexity. Second, due to lack of evidence on the effectiveness of alcohol intervention among HIV-infected patients in East Africa, the prior information was primarily based on one study [36], however, our VOI model structure allows for it to be updated if additional evidence becomes available. Third, decision uncertainty caused by cost variables was not addressed in this study.

In summary, we identified distributions of decision uncertainty regarding the adoption of alcohol interventions for HIV-infected patients in East Africa over a range of willingness-to-pay thresholds, quantified that uncertainty, and specified optimal sample sizes using a VOI approach rather than based on the minimum statistical power that is required to detect a pre-specified effect size. In situations in which trial information is likely to yield clinically actionable inferences with health importance, the VOI approach leads to larger sample sizes compared to power-based estimates, equivalent to requiring *p*-values below 0.05. In situations in which trial information is unlikely to yield clinically actionable inferences with health importance, the approach leads to smaller sample sizes compared to power-based estimates, equivalent to allowing p-values above 0.05. Systematic application of this approach to trial design questions would be expected to produce increased health benefit from available resources for conducting research.

## Conclusions

Value of information methods can be used to determine the optimal sample sizes of alcohol trials by reducing the risk of implementing less cost-effective alcohol strategies for HIV programs in East Africa, and they can be used as alternative approaches for the design of new trials when health care resources are limited. If reducing unhealthy alcohol use is a long-term goal for HIV programs in East Africa, additional new trials with optimal sample sizes ranging from 100 to 250 could save the opportunity cost of implementing less cost-effective alcohol strategies. Otherwise, conducting a new trial is not recommended.

## Abbreviations

ART: Antiretroviral therapy
ENBS: Expected net benefit of sampling
EVPI: Expected value of perfect information
EVSI: Expected value of sample information
HIV: Human immunodeficiency virus
NIH: National Institute of Health
QALY: Quality-adjusted life year
RCT: Randomized controlled trial
STIs: Sexually transmitted infections
VOI: Value of information
WTP: Willingness-to-pay
